# Data‐driven elemental descriptors for rational design of high‐sensitivity extreme ultraviolet photoresists

**DOI:** 10.1002/smo2.70056

**Published:** 2026-05-18

**Authors:** Jiyuan Liu, Jialiang Wei, Huie Zhu, Xiaojun Peng

**Affiliations:** ^1^ Zhangjiang Laboratory Shanghai China; ^2^ State Key Laboratory of Fine Chemicals Frontiers Science Center for Smart Materials School of Chemical Engineering Dalian University of Technology Dalian China

**Keywords:** data‐driven descriptors, EUV, high sensitivity, photoresists, rational design

## Abstract

The rational design of high‐sensitivity photoresists for extreme ultraviolet (EUV) lithography is hindered by the lack of quantitative, predictive descriptors that link material composition to EUV absorption. Here, we develop a density‐free, data‐driven approach that quantifies both elemental sensitivity and impact. We introduce elemental sensitivity descriptors, *μ*_*n*_ and *A*_*n*_, to estimate how elemental doping alters the linear attenuation coefficient and absorbance without requiring density information of the target material. Furthermore, we define elemental impact Δ*ϕ*_*i*_ to evaluate whether an element acts as a positive or negative contributor when embedded in high‐absorption compounds. We identify I, Te, In, Sn, Sb, Cs, and Bi as top candidates for EUV photoresists, while revealing the detrimental role of H and C as matrix components. This work provides a generalizable strategy for EUV photoresist design.

## Graphical abstract

Data‐driven descriptors indicate that elements such as I, Te, In, Sn, Sb, Cs and Bi have positive impact on EUV sensitivity in photoresists, whereas elements like C and H exhibit negative contributions.

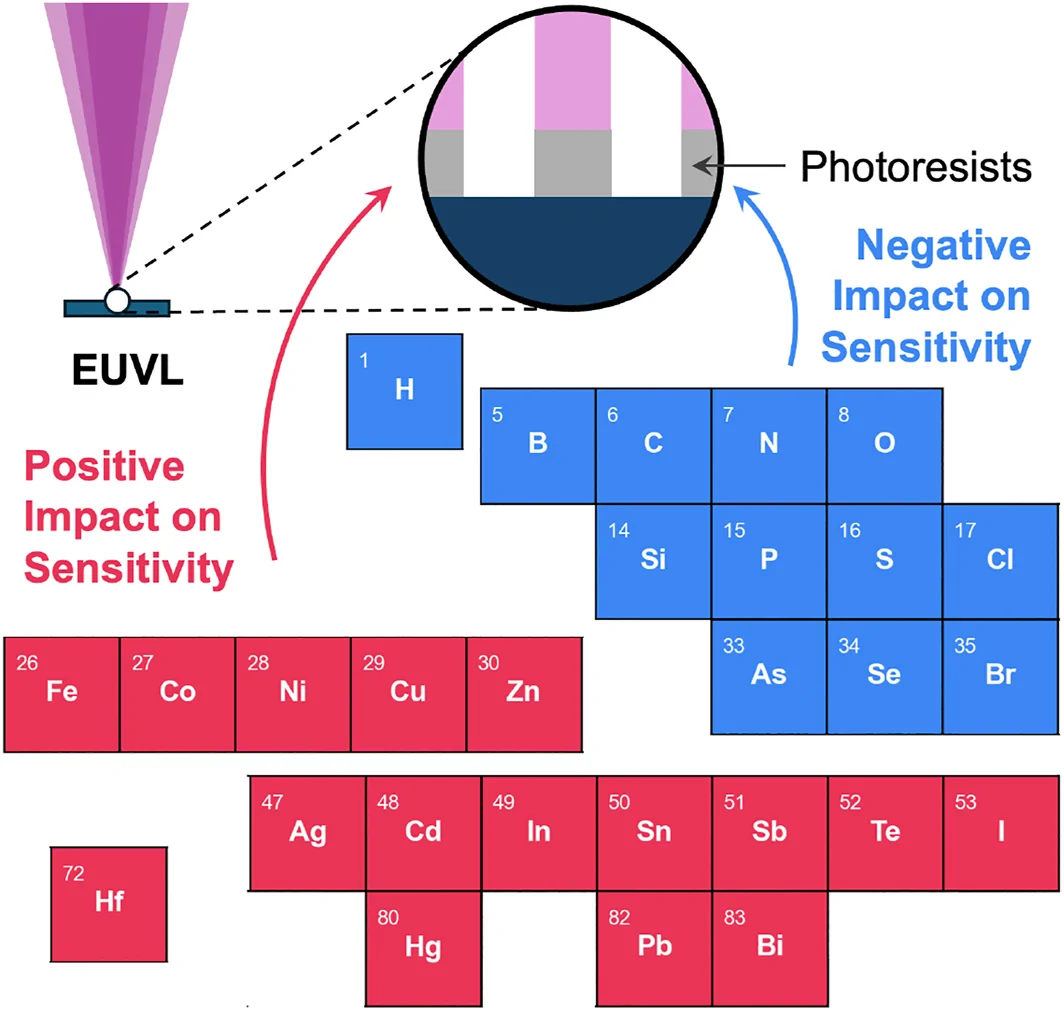

## 1 INTRODUCTION

The drive for more powerful and efficient computing continues to fuel innovation in semiconductor manufacturing. Extreme ultraviolet (EUV) lithography has emerged as the leading‐edge technology, successfully enabling feature sizes below 5 nm.^[1]^ EUV lithography relies on photoresists to absorb EUV light and precisely replicate mask patterns.[^2^, ^3^, ^4^, ^5^] Therefore, a photoresist's ability to efficiently absorb incident EUV light is paramount, directly impacting achievable resolution and manufacturing yield. Currently, chemically amplified resists (CARs) based on organic resins dominate the industry. However, these photoresists typically exhibit relatively low EUV light absorption, characterized by a low photoabsorption cross‐section and limited quantum yield.[^6^, ^7^, ^8^] Consequently, the development of novel photoresists with significantly enhanced EUV absorption capabilities is crucial.

The linear attenuation coefficient (*μ*), a key indicator of a material's ability to absorb light, typically ranges from 3 to 6 μm^−1^ for conventional organic photoresist resins.^[9]^ However, strategic material composition can significantly increase this value. As illustrated in Figure 1, incorporating certain metal elements, notably Sn, Hf, ionic salts and halogens (F, I), demonstrably enhances EUV light absorption.[^11^, ^12^, ^13^, ^14^, ^15^, ^16^] Metal salt sensitizers added to CAR formulations can increase *μ* by approximately 6%.^[17]^ Moreover, substituting hydrogen atoms with heavier halogens, like fluorine and iodine, within the resist structure can yield absorption coefficients exceeding 10 μm^−1^, as seen with halogenated polystyrene.^[9]^ This increase stems not only from the intrinsic absorption characteristics of the halogens but also from the corresponding increase in material density, a critical factor for efficient EUV absorption. Emerging metal‐inorganic resists offer even greater potential,^[18]^ with widely studied Hf‐oxo and Sn‐oxo resists demonstrating *μ* values reaching 9 and 14 μm^−1^.^[12]^ In addition, theoretical work on material density and energy gap calculation also remarks the effect on *μ* by ligands, counterions and doping, providing an alternative way to virtually evaluate the EUV sensitivity of photoresists.[^8^, ^19^] These results powerfully underscore the importance of *μ* as a crucial guiding principle in material component selection for advanced photoresist design.

**FIGURE 1:**
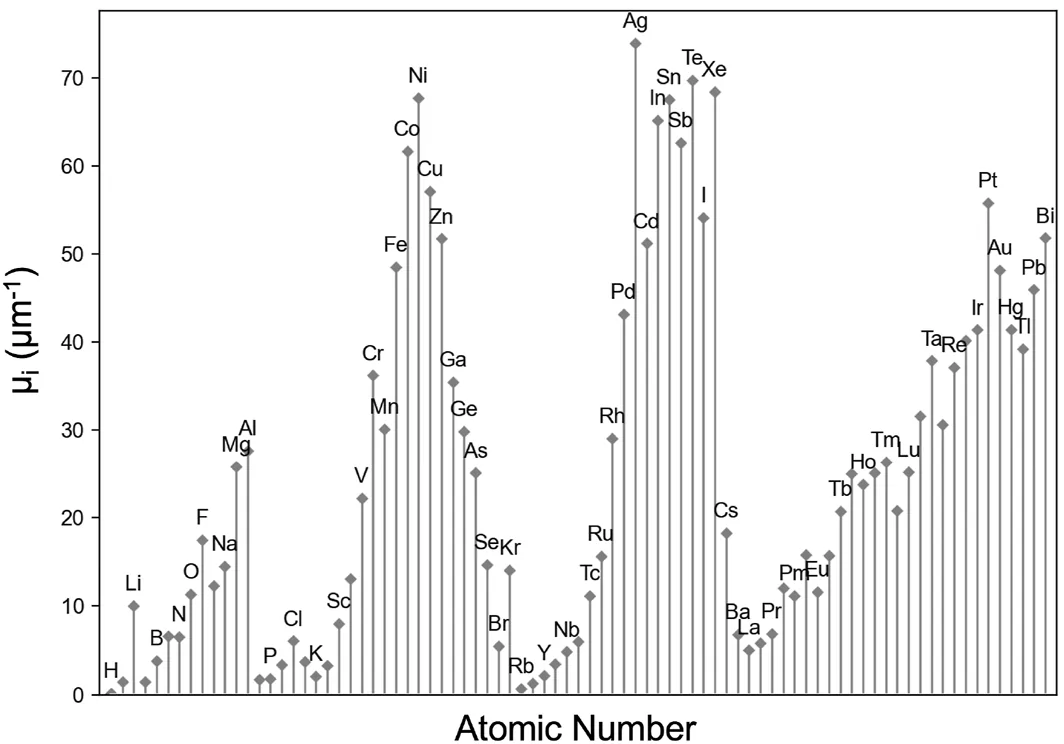
Linear attenuation coefficient *μ* (μm^−1^) for each element *i* in elements with atomic number *Z* = 1–83 under the incident light with a wavenumber of 13.5 nm. The data was collected from the CXRO (the center for X‐ray optics) database.^[10]^

Despite these promising advances, accurately predicting photoresist performance remains a significant challenge. Existing absorption data are largely limited to single‐component measurements, failing to capture the complex interplay of elements within a complete resist formulation. Calculating *μ* based solely on a material's chemical formula and density provides a basic understanding, but lacks the quantitative insight needed for effective material design and screening, as changes in chemical composition inevitably impact both *μ* and density.^[20]^ Consequently, a robust quantitative model capable of elucidating the contribution of each element to overall EUV absorption is critically needed to accelerate innovation in high‐sensitivity photoresists.

To address this challenge, we present a novel approach that leverages existing data to quantitatively reveal the sensitivity and impact of individual elements for EUV absorption. By integrating these data, we construct data‐driven descriptors that quantify the influence of elemental composition on EUV material absorption without any density information on target materials. This method overcomes the limitations of traditional qualitative analysis, providing a computationally efficient and predictive framework for designing the next generation of high‐sensitivity EUV photoresists and enabling continued advancements in semiconductor manufacturing.

## 2 THEORY

The mass attenuation coefficient *μ*/*ρ* of a compound *M* with molecular weight MW is expressed as

(1) $$\begin{array}{c} \frac{\mu_{M}}{\rho_{M}}=\frac{1}{MW}\;\Sigma_{i}\;AW_{i}n_{i}\frac{\mu_{i}}{\rho_{i}}\; \end{array}$$

where *AW*_*i*_ and *n*_*i*_ are the atomic weight and number of atoms for each element *i*, respectively. Since the molecular volume *V*_*M*_ and atomic volume *V*_*i*_ can be calculated as

(2) $$\begin{array}{c} V_{M}=\frac{MW}{\rho_{M}},V_{i}=\frac{AW_{i}}{\rho_{i}}\; \end{array}$$

the linear attenuation coefficient of compound *M*, *μ*_*M*_, can thus be calculated as

(3) $$\begin{array}{c} \mu_{M}=\frac{\Sigma_{i}n_{i}\mu_{i}V_{i}}{V_{M}}\; \end{array}$$

Based on the Equation (3), each element contribution *q*_*i*_ for *μ*_*M*_ is

(4) $$\begin{array}{c} q_{i}=\frac{n_{i}\mu_{i}V_{i}}{\mu_{M}V_{M}} \end{array}$$

The number of atoms for each element *i* will have impact on *μ*_*M*_ as

(5) $$\begin{array}{c} \mu_{n}=\frac{\partial \mu_{M}}{\partial n_{i}}=\frac{\mu_{i}V_{i}\;-\;\mu_{M}V_{i}^{x}}{\sum_{i}n_{i}V_{i}^{x}} \end{array}$$

Considering that changing the number of atoms in the chemical formula will change the *V*_*M*_ as well, *V*_*M*_ should be approximated as a function of *n*_*i*_

(6) $$\begin{array}{c} V_{M}=\Sigma_{i}^{m}n_{i}V_{i}^{x} \end{array}$$

where *V*_*i*_^*x*^ is the fitted atomic volume of specific materials, and this function is only applied when *n*_*i*_ is involved as a variable. The *μ*_*n*_ exhibits the number change influence in chemical formula on *μ*_*M*_, which directly reflects the intensity of elemental stoichiometric sensitivity.

Similarly, this descriptor can be applied to the transmission (T)

(7) $$\begin{array}{c} T=e^{-d\mu } \end{array}$$

(8) $$\begin{array}{c} T_{n}=\frac{\partial T_{M}}{\partial n_{i}}=\frac{V_{i}\;\ln\;T_{i}\;-\;V_{i}^{x}\;\ln\;T_{M}}{\sum_{i}n_{i}V_{i}^{x}}T_{M} \end{array}$$

and absorbance (A)

(9) $$\begin{array}{c} A=1\;-\;T \end{array}$$

(10) $$\begin{array}{c} A_{n}=\frac{\partial A_{M}}{\partial n_{i}}=-T_{n} \end{array}$$

Since *T* and *A* are as a function of the thickness of the materials, a common photoresist film of 30 nm was assumed in the following calculations. The *V*_*i*_^*x*^ can be directly fitted from the densities of materials in the database. These data‐driven descriptors were initially incorporated into the chemical formula and density of materials, enabling density‐free applications.

## 3 RESULTS AND DISCUSSION

To calculate the descriptors, we utilized a database of 908 small organic molecules from chemistry database (CSDB) in Shanghai Institute of Organic Chemistry,^[21]^ containing chemical formula and density data for elements C, H, O, F, Cl, Br, and I. The resulting values for *V*_*i*_^*x*^ were fitted with an *R* ^2^ of 0.97 and a mean absolute percentage error (MAPE) of 0.04 (Figure 2a). Figure 2b reveals the distribution of calculated *μ*_*n*_ values for each element, with yellow and red bars indicating the maximum and minimum values, the 90th and 10th percentile, respectively. The red line represents the 80% interval of the distribution, and the black rectangle marks the median *μ*_*n*_ value for each element. The measured *μ*_*M*_ values of a series of polystyrene and its derivatives (Table 1, Supporting Information S1: Figure S1) were collected to verify the descriptors.

**FIGURE 2:**
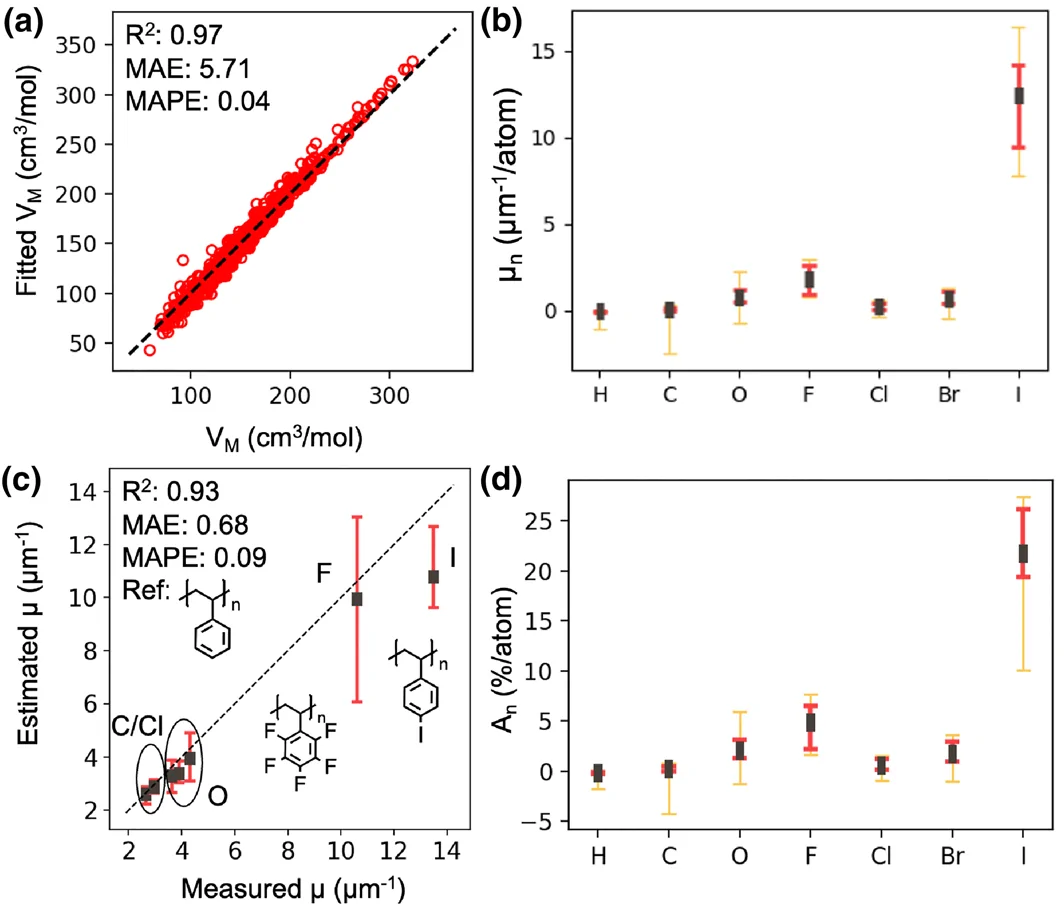
The elemental stoichiometric sensitivities for *μ* and A of organic molecules. (a) The fitting of *V*_*i*_^*x*^ in the chemistry database CSDB, where the values for each element are presented in the Supporting Information S1: Table S1. (b) The distribution of elemental stoichiometric sensitivities for *μ* (*μ*_*n*_). (c) The comparison between estimated and measured *μ* in Table 1. (d) The distribution of elemental stoichiometric sensitivities for A (*A*_*n*_). The yellow and red bars indicate the maximum and minimum values and the 90th and 10th percentiles of the distribution, respectively. The black rectangle marks the median *μ*_*n*_ value for each element.

**TABLE 1:** Measured linear attenuation coefficient of compound (*μ*_*M*_), estimated *μ*_*M*_, measured absorbance (A) and estimated A of various materials under 13.5 nm incident light.

Material	Measured *µ*_*M*_ (μm^−1^)	Estimated *µ*_*M*_ (μm^−1^)^a^	Measured *A*_*M*_ (%)	Estimated *A*_*M*_ (%)^a^
Polystyrene^b^	2.71	—	8.1	—
Poly(4‐hydroxystyrene)^b^	3.90	3.38	11.4	9.9
Poly(4‐methylstyrene)^b^	2.66	2.61	8.3	7.9
Poly(4‐chlorostyrene)^b^	2.97	2.88	9.0	8.5
Poly(pentafluorostyrene)^b^	10.61	9.95	24.0	25.6
Poly(4‐methoxystyrene)^b^	3.63	3.27	9.3	9.7
Poly(4‐acetoxystyrene)^b^	4.32	3.94	11.4	11.5
Poly(4‐iodostyrene)^b^	13.49	10.79	30.8	23.8
Tin‐OH^c^	10.9	—	—	—
Tin‐M^c^	14.7	11.66	—	—
Tin‐A^c^	14.2	11.09	—	—
Tin‐F^c^	11.6	12.65	—	—
Tin‐S^c^	11.1	10.90	—	—
Tin‐NO_3_ ^d^	11.45	11.65	—	—

*Note*: Structures can be found in Supporting Information S1: Figures S1 and S4.

^a^ The median values were set as the estimated results.

^b^ The measured data was collected from ref.^[9]^

^c^ The measured data was collected from ref.^[12]^

^d^ The measured data was collected from ref.^[22]^

The *μ*_*n*_ values incorporate molecular density and are normalized to the number of atoms in the chemical formula. Consequently, a larger *μ*_*n*_ value indicates a greater impact from changes in chemical composition. Iodine exhibits the most significant impact on *μ*_*M*_, while fluorine, oxygen, and bromine can make a noticeable difference. The contributions from other elements are negligible. Notably, negative contributions are also observed. The *μ*_*n*_ allows for direct estimation of light absorption effects such as iodine, for instance, by

(11) $$\begin{array}{c} \mu_{M}\left(C_{8}H_{7}I\right)=\mu_{M}\left(C_{8}H_{8}\right)-\mu_{n}\left(H\right)+\mu_{n}\left(I\right) \end{array}$$

Therefore, the *μ*_*M*_ of all polystyrene derivatives could be estimated, as shown in Figure 2c. The estimation achieved an *R* ^2^ of 0.93 and a MAPE of 0.09. This accurate estimation, validated through cross‐validation between the small organic molecule database trained descriptors and polymer properties, demonstrates the quantitative ability of *μ*_*n*_. The underestimation of *μ*_*n*_(I) may be attributed to scaling issues of iodine from small molecules to polymers. The wider 80% interval for poly(pentafluorostyrene) is caused by five times of the fluorination on hydrogen atom, which could accumulate errors.

Similarly, the elemental stoichiometric sensitivity for absorbance was also considered for 30 nm films (Figure 2d, Supporting Information S1: Figure S2b). A comparable trend, though with a slightly different distribution, was observed compared to the attenuation data. Iodine and fluorine appear to have the most significant impact on the estimated *A*_*M*_. Both *μ*_*n*_ and *A*_*M*_ can accurately estimate the corresponding property. The influence of thickness on the descriptor *A*_*n*_ was investigated (Supporting Information S1: Figure S2a), which does not alter the qualitative conclusion.

All elements up to bismuth (*Z* = 1–83) were also investigated to provide a widely covered guideline. The Cambridge Structural Database (CSD)^[23]^ was utilized for crystal property prediction and applied the following criteria to filter the data: (1) exclusion of elements with atomic numbers >83; (2) exclusion of noble gas elements; (3) crystal density between 0.4 and 5 g/cm^3^; (4) molecular volume between 1 and 20,000 cm^3^/mol; (5) absolute percentage error (APE) less than 0.1% between the molecular weights calculated by the formula and provided by the database; (6) APE less than 10% between calculated and database molecular volume; (7) exclusion of organic crystals containing only C, H, O, N, F, P, S, Cl, Br and I. Criteria 1–3) define the scope of normal photoresists, excluding those containing noble gases or radioactive elements. Criterion 4) mitigates numerical instability caused by extreme values, as molecular volume serves as the denominator in the calculation. Criteria 5–6) verify the consistency between the molecular formula, molecular weight and volume, ensuring data validity. Finally, criterion 7) excludes organic crystals, as more accurate data for these materials are provided in the preceding section. With this filter, 151,859 entries were utilized for *V*_*i*_^*x*^ fitting (Supporting Information S1: Figure S3). As the element Pm was not present in the filtered data, it was also removed from subsequent charts.

Figure 3a exhibits the statistical *μ*_*n*_ of elements in inorganic crystals, reflecting the elemental sensitivity for *μ* to doping or loading. The median of each elemental sensitivity value was used for subsequent discussion. Iodine exhibits the highest *μ*_*n*_ of 2.3 μm^−1^/atom, meaning that adding one additional iodine atom to the chemical formula will result in a 2.3 μm^−1^ increase in *μ*_*M*_, which is 10 times greater than that of fluorine. Notably, the *μ*_*n*_ of iodine in the crystal significantly decreases compared to the value in organic molecules (Figure 2b). This is attributable to the density distribution that the densities of most organic molecules range from 0.6 to 1 g/cm^3^, where additional iodine has a significant impact on density, whereas the densities of crystals rise to ∼1.5 g/cm^3^ (Supporting Information S1: Figure S3), resulting in a diminished impact on *μ*. Therefore, a specific database is crucial for accurately computing *μ*_*n*_. Some *μ*_*n*_ were also verified by the Sn‐oxo photoresists (Supporting Information S1: Figure S4) from different sources[^12^, ^22^] in Table 1, which estimate changes in *μ*_*M*_ when the original anion OH‐ is substituted with other anions.

**FIGURE 3:**
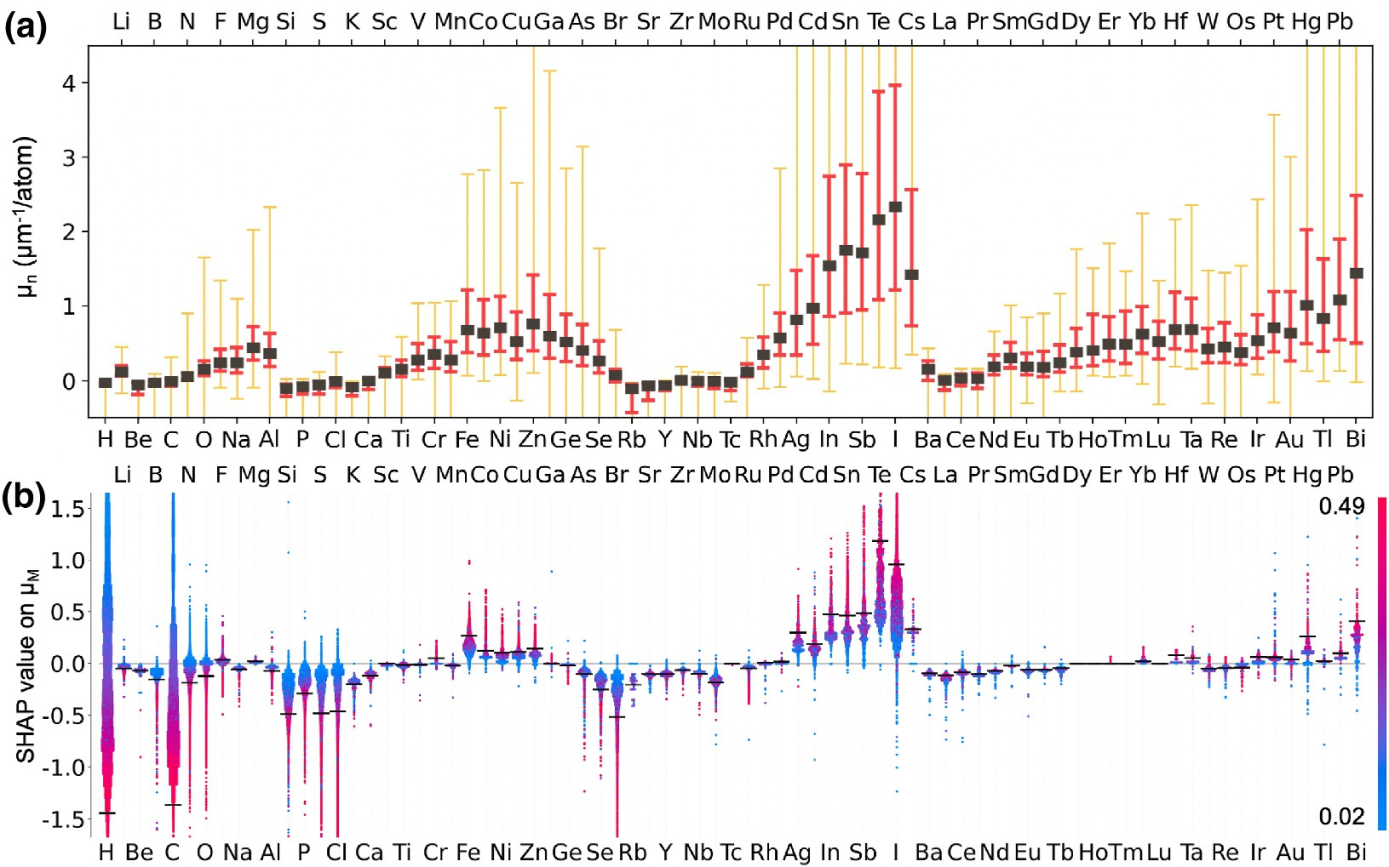
The elemental descriptors of inorganic crystals. (a) The elemental stoichiometric sensitivities for *μ* of inorganic crystals. (b) The elemental impact of inorganic crystals analyzed by Shapley additive explanation value. Each point marks the additive impact *ϕ*_*i*_ of *q*_*i*_ on the mean prediction of *μ*_*M*_, *ϕ*_0_, while the *q*_*i*_ value is reflected by the color. The black bar exhibits the elemental impact Δ*ϕ*_*i*_ for corresponding element.

Te exhibits competitive sensitivity as iodine, while In, Sn, Sb, Cs and Bi have similar sensitivity just below that of Te and I. More common 3d metals Fe, Co, Ni, Cu and Zn as well as 4p elements Ga, Ge and the 5d metal Hf have similar *μ*_*n*_, however, only 1/4 of that of I can contribute to the doping/loading system. Meanwhile, some elements, such as Si, P and S, even have negative contribution to the *μ*_*M*_ due to their poor cross sections or small densities.

Unfortunately, the elemental sensitivity only reflects the ability to tune *μ*_*M*_ for an existing compound of an element. It remains unclear which component can result in a high initial *μ*_*M*_ for a compound. Therefore, the elemental impact Δ*ϕ*_i_ was proposed to quantitively describe the positive or negative impact of each element on a compound as a component (Figure 3b). This can be calculated by

(12) $$\begin{array}{c} \Delta \phi_{i}=\phi_{i}^{50}+\phi_{i}^{75}-\phi_{i}^{25} \end{array}$$

where *ϕ*_*i*_ was the additive impact for each element calculated using the Shapley additive explanation (SHAP) TreeExplainer tool.^[24]^ The superscripts 75, 50 and 25 represent the 75th, 50th and 25th percentiles for normalized *q*_*i*_. The regression relationship between the element contribution *q*_*i*_ (Equation 4) and *μ*_*M*_ was interpreted using SHAP, where the feature matrix **Q** = [*q*_entry, *i*_] and true value matrix **M** = [(*μ*_*M*_)_entry_] were used to establish the relationship using XGBoost (Supporting Information S1: Figure S5). *ϕ*_*i*_ expresses the additive impact on mean prediction *ϕ*_0_ of 5.42 μm^−1^ in this case. Δ*ϕ*_*i*_ as a descriptor exhibits the value of increasing *q*_*i*_ impact on *ϕ*_0_, where positive Δ*ϕ*_*i*_ indicates a positive impact on *μ*_*M*_ as the component of a compound.

As shown in Figure 3b, a similar trend to the elemental sensitivity can be observed that Te and I have the highest positive impact on *μ*_*M*_ as main components in a compound, followed by the highly sensitive elements In, Sn, Sb, Cs and Bi. Conversely, H and C have the highest negative impact on *μ*_*M*_, meaning high *q*_*H*_ or *q*_*C*_ of a compound will result in a *μ*_*M*_ lower than 5.42 μm^−1^. Si, S, Cl and Br also exhibit a high negative impact on a compound. It is notable that C and H are fundamental components in inorganic photoresist, playing important roles in crosslinking to form resist matrices. The nature to cause high *q*_*H*_ or *q*_*C*_ of a compound is the relatively low *q*_*i*_ from other elements. Therefore, high positive impact elements are necessary for high EUV‐absorbed photoresist. The overall elemental sensitivity and importance of inorganic crystals for EUV absorbance are summarized in the Figure 4.

**FIGURE 4:**
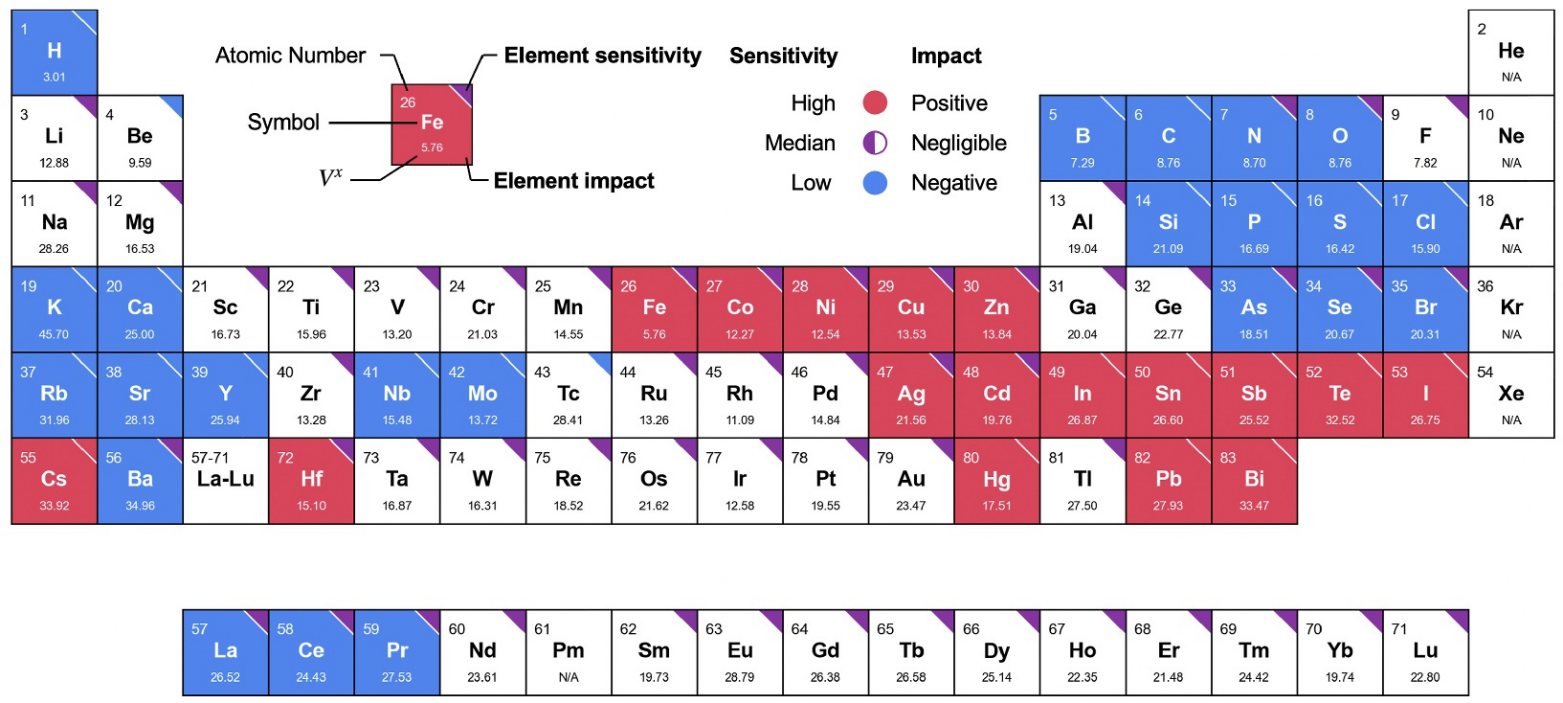
The elemental sensitivity and impact of inorganic crystals on EUV absorbance.

Several potential factors affect the accuracy of these descriptors. As the above cases reveal, there is a significant difference in elemental sensitivity between organic polymers and inorganic crystals. A specific database of chemical formulas and accurate densities in related fields is necessary for accuracy. Furthermore, accurate photoabsorption cross sections for elements are also important. Some elements have been reported to require remeasuring due to mismatches in absorption energy levels under EUV.[^25^, ^26^] As this approach to evaluate elemental sensitivity and importance is universal and covers both EUV and X‐rays, obtaining suitable elemental cross sections is a challenging task.

## 4 CONCLUSION

In summary, the data‐driven elemental sensitivity and impact descriptors have been proposed in this work. The elemental stoichiometric sensitivity for attenuation coefficient (*μ*_*n*_) and absorbance (*A*_*n*_) are demonstrated for estimating the loading/doping elemental sensitivity of compound for *μ*_*M*_ and *A*_*M*_ in organic polymers. I, F and O are promising elements for modifying the polymers to improve the sensitivity. The elemental sensitivity and impact (Δ*ϕ*_*i*_) of inorganic crystals were also calculated. Te, I as well as In, Sn, Sb, Cs and Bi are expected to exhibit high elemental sensitivity and positive impact on *μ*_*M*_, while H and C are supposed to have negative impact on *μ*_*M*_, indicating the lack of elements with positive impact. The sensitivity descriptors quantify how doping or changing the number of atoms of a specific element alters the linear attenuation coefficient, estimating the sensitivity of a material to compositional changes. The impact descriptor evaluates whether an element acts as a positive or negative contributor when embedded as a main component within a compound. It is used to determine which elements result in a high initial absorption for a compound. These density‐free descriptors will serve as guidance for EUV photoresist design.

## CONFLICT OF INTEREST STATEMENT

The authors declare no conflicts of interest.

## ETHICS STATEMENT

No animal or human experiments were involved in this study.

## Supporting information

Supporting Information S1

## Data Availability

The data that support the findings of this study are available on request from the corresponding author. The data are not publicly available due to privacy or ethical restrictions.
