# Supplementary material for: Data‐driven elemental descriptors for rational design of high‐sensitivity extreme ultraviolet photoresists
Source: Smart Mol. 2026 May 18:e70056. Online ahead of print. doi: 10.1002/smo2.70056 (PMC13398669; doi:10.1002/smo2.70056)
Supplement: Supplementary file 1 — Supporting Information S1 [file SMO2-9999-0-s001.docx]

**Supplementary Information**

**Data-Driven Elemental Descriptors for Rational Design of High-sensitivity Extreme Ultraviolet Photoresists**

Jiyuan Liu^1^, Jialiang Wei^1^, Huie Zhu^1^* and Xiaojun Peng^1,2^

*^1^Zhangjiang laboratory, Shanghai 201210, P. R. China;*

*^2^State Key Laboratory of Fine Chemicals, Frontiers Science Center for Smart Materials, School of Chemical Engineering, Dalian University of Technology, 2 Linggong Road, Dalian 116024, P. R. China*

Corresponding Authors: Huie Zhu([zhuhe@zjlab.ac.cn](mailto:zhuhe@zjlab.ac.cn))


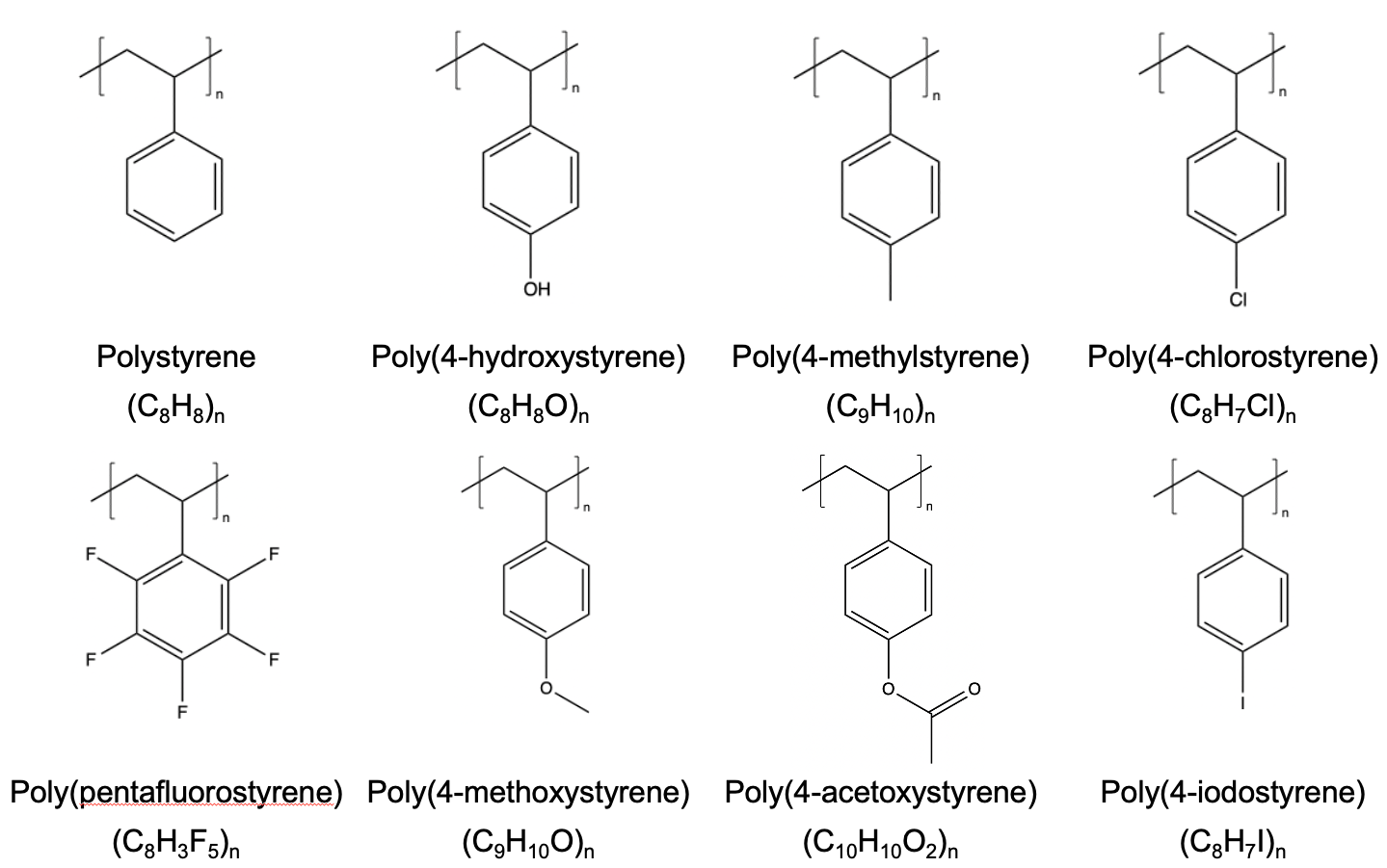


**Figure S1** The structures of polymers in Table 1.


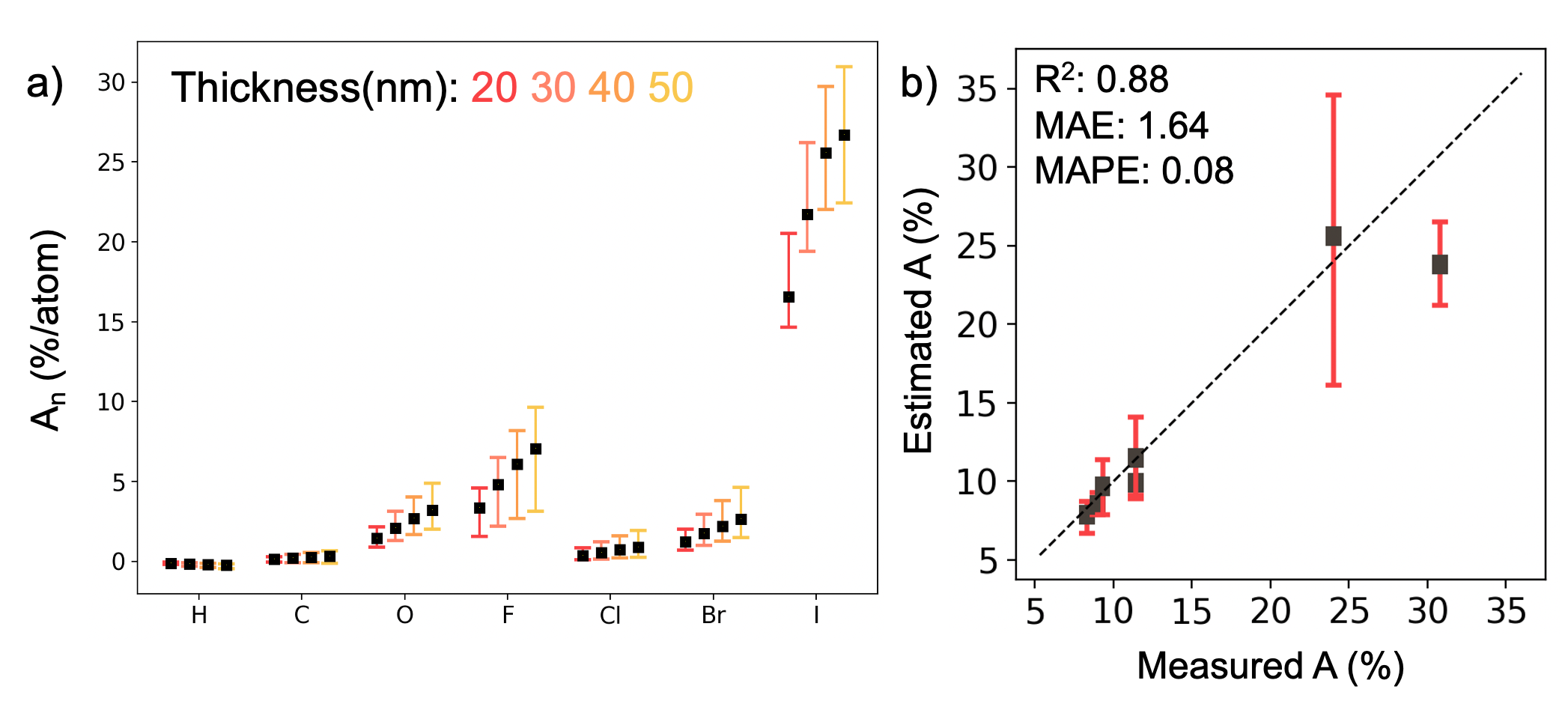


**Figure S2** a) The distribution of elemental stoichiometric sensitivities for A (A_n_), considering compounds with different thicknesses. The bars indicate the 90th and 10th percentile of the distribution, where different colors represent different thicknesses. The black rectangle marks the median A_n_ value for each element. b) The comparison between estimated and measured A in Table 1.


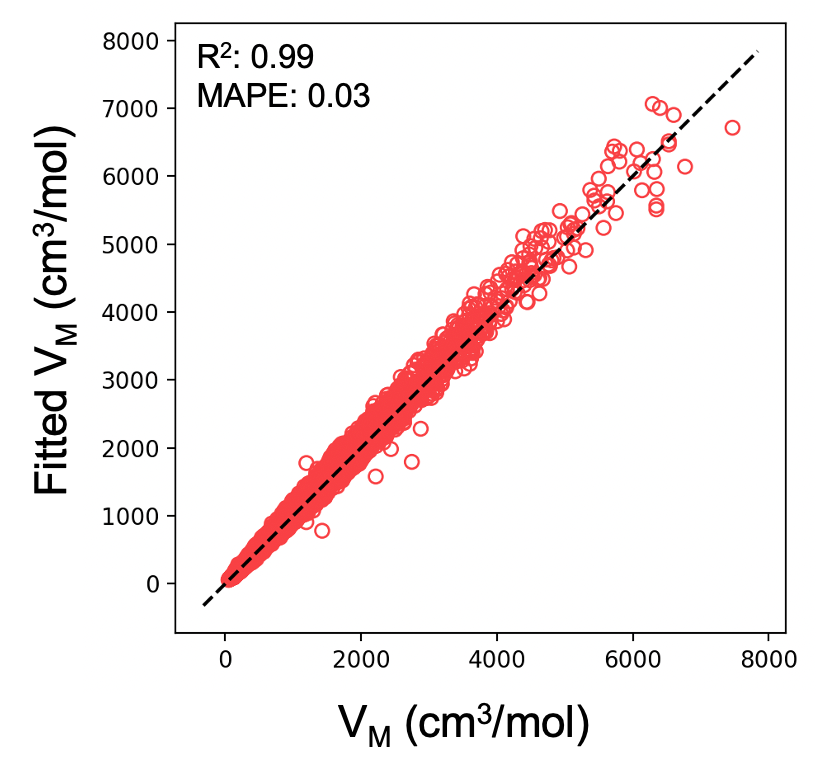

**Figure S3** The fitting of V^x^ in the filtered Cambridge structure database (CSD).


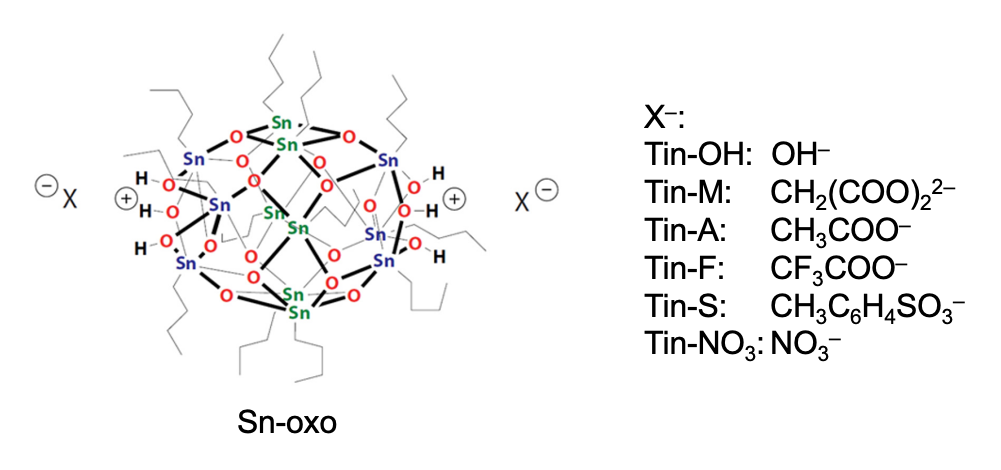

**Figure S4** The structures of Sn-oxo photoresists in Table 1.


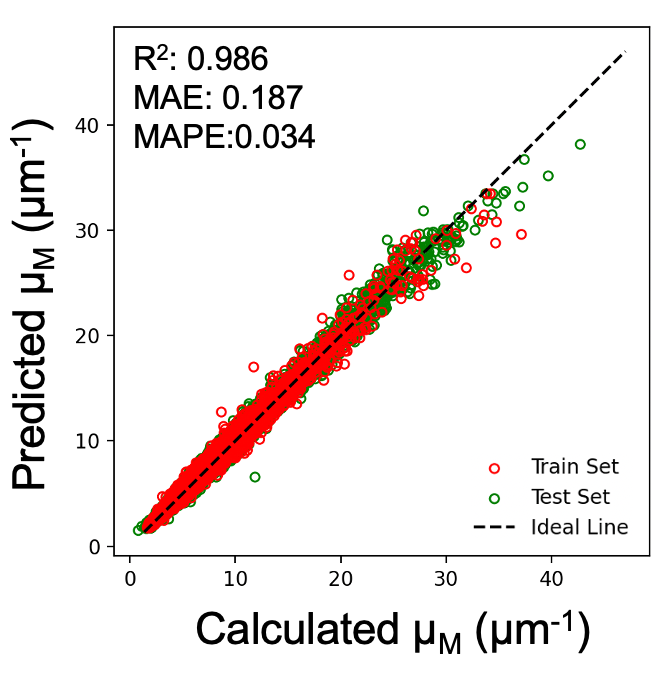


**Figure S5** The comparison between predicted and calculated µ_M_. The µ_M_ was predicted by XGBoost model considering q_i_ matrix as input feature.

**Table S1.** The V_i_^x^ of organic molecules.

| Elements | H | C | O | F | Cl | Br | I |
| --- | --- | --- | --- | --- | --- | --- | --- |
| V_i_^x^(cm^3^/mol) | 5.00 | 8.14 | 8.11 | 11.15 | 19.92 | 25.00 | 35.19 |
